# Family planning service receipt during facility visits in Ethiopia: Evidence from the 2021–2022 service provision assessment survey

**DOI:** 10.1371/journal.pone.0352145

**Published:** 2026-07-09

**Authors:** Melkamu Chafamo Joche, B. Muniswamy, B. Punyavathi

**Affiliations:** 1 Department of Statistics, College of Natural and Computational Science, Wachemo University, Hosanna, Ethiopia; 2 Department of Statistics, College of Science and Technology, Andhra University, Visakhapatnam, Andhra Pradesh, India; Haramaya University, ETHIOPIA

## Abstract

**Background:**

Family planning is a central component of reproductive health and contributes to reductions in maternal and child mortality. While most evidence in Ethiopia is derived from household surveys, limited attention has been given to service delivery at the point of care. This study examines determinants of receipt of family planning services during facility visits using nationally sampled Service Provision Assessment (SPA) survey data.

**Methods:**

A facility-based cross-sectional analysis was conducted using nationally implemented SPA survey data. The survey employed a stratified multistage sampling design, in which health facilities were selected within strata defined by region, managing authority, and facility type, followed by systematic sampling of clients within facilities for exit interviews. The study included 2,588 women aged 15–49 years who attended sampled facilities and completed client exit interviews. The outcome was receipt of any family planning service during the visit, defined as provision of counseling and/or a contraceptive method, reflecting service delivery at the point of care rather than contraceptive use. Multivariable logistic regression was used to assess associations between client characteristics, provider attributes, and facility-level factors. Exploratory interaction analyses were conducted but not retained in the final model due to instability and sparse data structures. Adjusted odds ratios (AORs) with 95% confidence intervals (CIs) were reported, and statistical significance was defined at a two-sided p-value < 0.05. All analyses were conducted using R (version 4.5.3).

**Results:**

Overall, 64.2% of clients received a family planning service during their visit. After adjustment, clients without prior contact with a provider had higher odds of receiving a service compared with those reporting prior contact (AOR = 2.25; 95% CI: 1.86–2.72; p < 0.001). Married women had lower odds of receiving a service than single women (AOR = 0.69; 95% CI: 0.49–0.98; p = 0.030). Substantial regional variation was observed, with lower odds in Amhara (AOR = 0.29; 95% CI: 0.17–0.49) and Addis Ababa (AOR = 0.41; 95% CI: 0.23–0.73), and higher odds in Benishangul-Gumuz, Gambella, and Dire Dawa. Other covariates were not statistically significant after adjustment, and observed associations should be interpreted as reflecting service delivery processes at the point of care rather than underlying contraceptive demand.

**Conclusions:**

Family planning service receipt during facility visits in Ethiopia is associated with prior client–provider contact, marital status, and regional context. The persistence of regional differences after adjustment indicates that contextual factors related to health system performance and service delivery environments play a role in shaping service delivery during clinical encounters. The findings indicate heterogeneity in service delivery at the point of care, suggesting the need for context-specific strategies to improve the consistency and quality of family planning services within health facilities.

## Introduction

Family planning (FP) is a core component of reproductive health, enabling individuals to achieve the desired fertility and spacing of births through the use of contraceptive methods. Extensive evidence demonstrates that contraceptive use reduces maternal mortality, prevents unintended pregnancies, and improves child health outcomes, particularly in low- and middle-income countries (LMICs) [1,2].

Despite these benefits, the utilization of family planning services remains uneven. Both demand-side factors such as fertility preferences, sociocultural norms, and knowledge and supply-side factors, including service availability, provider competence, and quality of care contribute to this variation [3,4]. Increasingly, research highlights that the quality of care and the nature of provider–client interactions play a critical role in shaping contraceptive uptake and continuation, beyond mere access to services [5–7].

Ethiopia has made substantial progress in expanding access to family planning through the strengthening of primary healthcare and the Health Extension Program. National surveys indicate increasing contraceptive prevalence over time; however, marked regional disparities persist, reflecting heterogeneity in health system performance, access to services, and sociocultural context [8]. Policy initiatives such as the Family Planning Costed Implementation Plan (FP-CIP 2023–2030) aim to address these gaps by improving service coverage and reducing unmet need [9].

Most existing evidence in Ethiopia is derived from household surveys, particularly the Demographic and Health Surveys (EDHS), which measure self-reported contraceptive use and emphasize individual- and household-level determinants [10,11]. While these data are essential for monitoring population-level outcomes, they fail to capture whether family planning services are actually delivered during facility visits and do not provide detailed information on provider behavior and service delivery processes at the point of care.

Facility-based data provide a complementary perspective. The Ethiopian Service Provision Assessment (SPA) survey integrates facility audits, provider interviews, and client exit interviews, enabling measurement of reported service receipt at the point of care [12]. This design allows examination of how client characteristics, provider attributes, and facility-level factors jointly influence whether family planning services are delivered during a visit an outcome that is conceptually distinct from population-level contraceptive use [13]. Regional variation in health system capacity, provider practices, and service environments in Ethiopia suggests that service delivery processes may differ across contexts. These differences provide a theoretical basis for examining whether associations between client characteristics and service receipt vary across regions and facility settings, even when overall service availability is similar [14].

However, empirical evidence using SPA data to examine factors influencing the receipt of family planning services in Ethiopia remains limited. In particular, there is a need to better understand how client, provider, and facility-level factors are associated with the delivery of services during facility visits and how these relationships reflect broader health system performance.

Therefore, this study uses nationally implemented SPA survey data to examine the factors that influence the receipt of family planning services during facility visits in Ethiopia. Specifically, the study aims to identify client, provider, and facility-level factors associated with receipt of family planning services during the visit and assess the extent of regional variation in service delivery. By focusing on observed service delivery at the point of care, the study provides a proximal measure of health system performance and contributes to a more precise understanding of how service delivery processes influence the receipt of family planning within health facilities.

## Materials and methods

### Study design and data source

This study employed a facility-based cross-sectional design using data from the 2021–2022 Ethiopian Service Provision Assessment (SPA) survey conducted under the Demographic and Health Surveys (DHS) Program. The SPA survey is a nationally implemented health facility assessment designed to capture information on service availability, readiness, and quality across public and private health facilities. The survey combines different types of data, such as inventories of facilities, interviews with health providers, and exit interviews with clients. The present analysis was based exclusively on the dataset from client exit interviews, which provides standardized information on services received during a single facility visit and on client provider interactions observed at the point of care. The SPA dataset was obtained from the DHS Program following standard procedures. All data are fully anonymized, and no personal identifiers are available to researchers.

### Study setting

The study covered health facilities across all administrative regions of Ethiopia, including both public and private institutions. The dataset reflects diverse geographic, socioeconomic, and health system contexts, ensuring broad representativeness of service delivery environments.

### Study population

The study population consisted of women aged 15–49 years who attended sampled health facilities and were included in the SPA client exit interview at facilities offering family planning services. The inclusion reflects attendance at facilities where FP services were available and participation in the exit interview. Only one observation per client visit was included, consistent with the SPA design, where each exit interview corresponds to a single service encounter. A total of 2,588 women were included in the analysis, of whom 1,662 (64.2%) received at least one family planning service during their facility visit.

### Sampling procedure

The Ethiopian SPA survey employed a stratified multistage sampling design. Health facilities were stratified by region, managing authority (public or private), and facility type (hospital, health center, clinic, and health post). Facilities were then randomly selected within strata to ensure nationally implemented in service delivery environments. Within each selected facility, eligible clients were systematically sampled on the day of data collection and interviewed immediately after receiving services. This approach minimizes recall bias and ensures that reported services correspond to actual care received during the observed visit.

The regression analysis did not include sampling weights or complex survey design elements like clustering and stratification. The data from client exit interviews represent facility-based service encounters rather than a population-based sample of women. Accordingly, the analysis focuses on associations within observed service delivery settings. Estimates may be subject to bias in variance estimation and generalizability, and findings should not be interpreted as nationally representative.

### Study variables

#### Outcome variable.

The outcome variable was defined as receipt of a family planning (FP) service during the facility visit, based on data from client exit interviews in the Service Provision Assessment (SPA) survey. It was coded as a binary variable (Yes/No). A client was classified as having received an FP service if, during the visit, the client reported receiving FP counseling or information and/or a contraceptive method, prescription, or referral.

Within the SPA framework, this measure reflects observed service delivery at the point of care rather than confirmed adoption, continuation, or sustained use of contraception. The outcome combines distinct service components, including counseling-only encounters and method provision. For fertility-awareness–based (natural) methods, “receipt” reflects counseling or information provision rather than physical delivery of a method. Although this composite definition follows the SPA measurement structure, it aggregates service components that may have different underlying determinants. Therefore, findings should be interpreted as reflecting overall service delivery at the visit rather than specific contraceptive behaviors such as initiation, continuation, or adherence.

#### Explanatory variables.

Explanatory variables were selected based on theoretical relevance to health service utilization and availability within the SPA dataset. These included client-level characteristics (educational status, marital status, number of pregnancies, and place of residence), provider-level characteristics (sex of the provider), and facility/service-related characteristics (facility type, prior client–provider contact, and region).

Categorical variables were coded using appropriate reference categories to facilitate interpretation of regression coefficients. Sparse categories, particularly within marital status and regional classifications, were carefully evaluated; however, categories were retained where analytically meaningful and supported by sufficient sample size to avoid over-collapsing and loss of interpretability.

### Statistical analysis

All analyses were conducted using R statistical software (version 4.5.3). Descriptive statistics were first computed to summarize the distribution of study variables. Categorical variables were reported as frequencies and percentages, while continuous variables were summarized using means and standard deviations or medians and interquartile ranges, as appropriate.

Descriptive analyses were conducted using unweighted data and did not incorporate clustering, stratification, or sampling weights because the primary objective was to examine conditional associations between client, provider-, and facility-level characteristics and receipt of family planning services during facility visits rather than to estimate nationally representative prevalence measures. Accordingly, the analysis followed a model-based inferential framework focused on within-sample associations using observed SPA facility-visit data. Methodological literature has emphasized that the appropriateness of survey weighting in regression analyses depends on the analytic objective and inferential target. Solon et al. noted that weighting is primarily required for descriptive population estimation and may not be necessary when the objective is estimation of conditional relationships and relevant design-related covariates are included in the model [15]. Similarly, Beaumont argued that inclusion of variables associated with the sampling process may reduce the need for design weights in regression analyses of conditional relationships [16]. Additional methodological work has further indicated that weighted regression models can reduce statistical efficiency when the primary objective is association modeling rather than descriptive population inference [17,18]. Consistent with these recommendations, major design-related covariates, including region, facility type, residence, and provider characteristics, were incorporated directly into the regression model to account for contextual heterogeneity. Nevertheless, findings should be interpreted as estimates of associations within the analyzed SPA facility-visit sample rather than nationally representative population parameters.

Differences in characteristics between clients who did and did not receive family planning (FP) services during the visit were assessed using Pearson’s chi-square tests for categorical variables and Wilcoxon rank-sum tests for continuous variables. To complement hypothesis testing, effect sizes were reported using Cramér’s V for categorical variables and rank-biserial correlation for continuous variables.

Standardized mean differences (SMDs) were additionally calculated to assess covariate imbalance between outcome groups. For continuous variables, SMDs were computed by standardizing mean differences using pooled standard deviations, whereas for categorical variables multivariate extensions based on category proportions were applied. SMDs are recommended as scale-independent measures of group imbalance because they are less influenced by sample size than conventional significance tests. Consistent with commonly used recommendations in observational and epidemiologic research, SMD values around 0.10, 0.20, and 0.50 were interpreted as indicating small, moderate, and large imbalance, respectively [19–21].

A multivariable logistic regression model was fitted to examine the association between explanatory variables and receipt of FP services during the visit. Adjusted odds ratios (AORs) with 95% confidence intervals (CIs) were reported. Statistical significance was assessed using a two-sided alpha level of 0.05.

The model specification was defined as:


$$\begin{array}{c} \text{logit}\left(\text{P}\left({\text{Y}}_{\text{i}}=\text{1}\right)\right)=\beta_{\text{0}}+\sum_{\text{k}=\text{1}}^{\text{p}}\beta_{\text{k}}{\text{X}}_{\text{ki}} \end{array}$$

where $Y_{i}$ represents the binary outcome for the client i, $X_{ki}$ represents the k-th explanatory variable, and $\beta_{k}$ represents regression coefficients.

Selection of covariates was guided by theoretical relevance to health service utilization and data availability within the SPA framework. Interaction terms between prior client–provider contact and contextual variables (region and facility type) were explored based on hypothesized effect heterogeneity. However, these interaction models exhibited substantial multicollinearity and sparse-data structures, leading to unstable parameter estimates and reduced interpretability. Therefore, the final analysis is based on the main-effects model.

Model diagnostics were conducted to assess the adequacy of the fitted model. Multicollinearity was evaluated using variance inflation factors (VIF), with values below 5 indicating no evidence of problematic collinearity, consistent with commonly applied recommendations in regression modeling studies [22]. The potential influence of individual observations was examined using Cook’s distance and leverage diagnostics.

Model fit was assessed using deviance statistics and the Akaike Information Criterion (AIC) to compare alternative specifications [23]. Discriminatory ability was evaluated using the area under the receiver operating characteristic curve (AUC), and calibration was assessed using the Brier score and calibration plots. These measures were interpreted as indicators of predictive performance and model stability, rather than evidence of causal validity. To enhance interpretability, average marginal effects (AMEs) were computed to quantify the change in predicted probability of receiving FP services associated with each explanatory variable, holding other variables constant [24,25].

Given the potential for endogeneity, particularly for prior client–provider contact, which may proxy unobserved characteristics such as underlying demand for family planning, all estimated associations are interpreted as non-causal. Results are reported in accordance with established guidelines for observational studies to ensure transparency and reproducibility.

### Ethical considerations

The study uses publicly available SPA data obtained with authorization from the DHS Program. Ethical approval for the original survey was obtained by the Ethiopian Public Health Institute Institutional Review Board and the ICF Institutional Review Board, and the survey was conducted in accordance with the ethical principles outlined in the Declaration of Helsinki. Permission to access and use the SPA 2021–2022 data was formally obtained from the DHS Program following an approved data request. The dataset used for this analysis is fully anonymized and publicly available; therefore, no additional ethical approval or informed consent was required for the present study.

## Results

### Study population and outcome distribution

A total of 2,588 women aged 15–49 years who attended sampled health facilities were included in the analysis. Among these, 1,662 (64.2%) reported receiving a family planning (FP) service during the visit, while 926 (35.8%) did not receive any FP service.

### Descriptive characteristics of study participants

**Table 1** presents the distribution of study participants according to receipt of family planning (FP) services during the facility visit. Several variables, including educational status, number of pregnancies, provider sex, and place of residence, showed minimal imbalance between outcome groups (standardized mean difference [SMD] < 0.10).

**Table 1:** Characteristics of study participants by family planning service receipt.

Characteristic	Overall(N = 2,588)	No (n = 926)	Yes (n = 1,662)	p-value	SMD
Ever attended formal school				0.60	0.03
Yes	1,913 (73.9%)	691 (74.6%)	1,222 (73.5%)		
No	675 (26.1%)	235 (25.4%)	440 (26.5%)		
Number of pregnancies, mean (SD)	2.27 (1.7)	2.23 (1.71)	2.29 (1.8)	0.60	0.03
Facility type				<0.001	0.19
Hospital	1,385 (53.5%)	444 (48.0%)	941 (56.6%)		
Health Center	837 (32.3%)	332 (35.9%)	505 (30.4%)		
Clinic	250 (9.7%)	111 (12.0%)	139 (8.4%)		
Health Post	116 (4.5%)	39 (4.2%)	77 (4.6%)		
Provider sex				0.11	0.07
Male	974 (37.6%)	368 (39.7%)	606 (36.5%)		
Female	1,614 (62.4%)	558 (60.3%)	1,056 (63.5%)		
Previous contact with provider				<0.001	0.32
Yes	1,776 (68.6%)	547 (59.1%)	1,229 (74.0%)		
No	812 (31.4%)	379 (40.9%)	433 (26.0%)		
Marital status				0.01	0.14
Single	186 (7.2%)	87 (9.4%)	99 (6.0%)		
Married	2,359 (91.2%)	823 (88.9%)	1,536 (92.4%)		
Widowed	12 (0.5%)	6 (0.6%)	6 (0.4%)		
Divorced	21 (0.8%)	8 (0.9%)	13 (0.8%)		
Separated	10 (0.4%)	2 (0.2%)	8 (0.5%)		
Region				<0.001	0.63
Afar	82 (3.2%)	39 (4.2%)	43 (2.6%)		
Amhara	428 (16.5%)	81 (8.7%)	347 (20.9%)		
Oromia	748 (28.9%)	251 (27.1%)	497 (29.9%)		
Somali	63 (2.4%)	17 (1.8%)	46 (2.8%)		
Benishangul-Gumuz	87 (3.4%)	62 (6.7%)	25 (1.5%)		
S.N.N.P.	473 (18.3%)	147 (15.9%)	326 (19.6%)		
Gambella	158 (6.1%)	111 (12.0%)	47 (2.8%)		
Harari	28 (1.1%)	11 (1.2%)	17 (1.0%)		
Addis Ababa	168 (6.5%)	44 (4.8%)	124 (7.5%)		
Dire Dawa	62 (2.4%)	43 (4.6%)	19 (1.1%)		
Sidama	291 (11.2%)	120 (13.0%)	171 (10.3%)		
Residence				0.20	0.05
Urban	1,610 (62.2%)	561 (60.6%)	1,049 (63.1%)		
Rural	978 (37.8%)	365 (39.4%)	613 (36.9%)		

In contrast, greater covariate imbalance was observed for prior client–provider contact (SMD = 0.32) and region (SMD = 0.63), indicating meaningful differences between women who did and did not receive FP services during the visit. Facility type (p < 0.001) and marital status (p = 0.01) also differed between groups, although the magnitude of imbalance was comparatively smaller.

Women who received FP services were more frequently observed in hospitals than women who did not receive FP services. Prior client–provider contact was descriptively more common among women who received FP services (74.0%) than among those who did not receive services (59.1%). However, this unadjusted distribution differed from the direction of the adjusted association observed in the multivariable model, indicating that the association appeared to vary according to the distribution of other covariates across groups. Marked regional variation in service receipt was also observed.

### Bivariate associations and effect size assessment

**Table 2** summarizes bivariate associations between explanatory variables and receipt of FP services during the visit. Statistically significant associations were observed for facility type (χ² = 22.17, p < 0.001) and prior client–provider contact (χ² = 60.43, p < 0.00). Marital status was also associated with the outcome based on Fisher’s exact test (p = 0.01). No statistically significant associations were identified for educational status (p = 0.57), provider sex (p = 0.11), place of residence (p = 0.20), or number of pregnancies (p = 0.56).

**Table 2:** Bivariate associations with family planning service receipt.

Variable	Test Statistic	p-value	Effect Size
Facility type	χ² = 22.17	<0.001	0.09
Previous contact	χ² = 60.43	<0.001	0.15
Marital status	—	0.01	—
Education	χ² = 0.32	0.57	—
Provider sex	χ² = 2.59	0.11	—
Residence	χ² = 1.64	0.20	—
Number of pregnancies	W = 759,058.00	0.56	0.01

*W=Mann–Whitney U statistic*

Effect size estimates indicated a moderate association for prior client–provider contact (Cramér’s V = 0.15) and a small association for facility type (Cramér’s V = 0.09), while the effect size for number of pregnancies was negligible (rank-biserial correlation = 0.01).

### Regional variation in family planning service receipt

Region was summarized separately to improve readability of the primary bivariate comparison table and to avoid disproportionate expansion of the table due to the large number of regional categories. Regional variation in receipt of FP services during facility visits is presented in **Table 3**. A statistically significant association was observed between region and service receipt (χ² = 236.60, df = 10, p < 0.001), with a moderate effect size (Cramér’s V = 0.30), indicating substantial variation across regions.

**Table 3:** Association between region and receipt of family planning services during facility visits.

Statistic	Value
χ²	236.60
Degrees of freedom	10
p-value	<0.001
Cramér’s V	0.30

**Table 4** present crude association between previous contact and FP service receipt. In unadjusted analysis, women without prior client–provider contact had significantly lower odds of receiving FP services during the visit compared with women who had prior contact (crude OR = 0.51; 95% CI: 0.43–0.60; p < 0.001). However, after adjustment for region, facility type, marital status, and other covariates, the direction of association reversed in the multivariable model.

**Table 4:** Crude association between previous contact and FP service receipt during facility visits.

Variable	Category	Crude OR	95% CI	p-value
Previous contact with provider	Yes (Ref.)	—	—	—
	No	0.51	0.43–0.60	<0.001

### Multivariable logistic regression analysis

**Table 5** presents adjusted associations between explanatory variables and receipt of FP services during the visit. After adjustment for region, facility type, marital status, and other covariates, the direction of association reversed, with women without prior contact demonstrating higher adjusted odds of receiving FP services during the visit (AOR = 2.25; 95% CI: 1.86–2.72; p < 0.001). This direction differed from the descriptive distribution observed in **Table 1**, suggesting that adjustment for contextual and facility-related covariates reversed the direction of the association. Marital status was also associated with service receipt. Married women had lower odds of receiving FP services compared with single women (AOR = 0.69; 95% CI: 0.49–0.98; p = 0.03), while other marital categories were not statistically significant.

**Table 5:** Multivariable logistic regression of factors associated with family planning service receipt.

Variable	Category	AOR	95% CI	p-value
Ever attended school	Yes (Ref.)	—	—	—
	No	0.92	0.74–1.14	0.47
Number of pregnancies	Continuous	1.02	0.97–1.08	0.43
Facility type	Hospital (Ref.)	—	—	—
	Health Center	1.18	0.96–1.44	0.13
	Clinic	1.06	0.77–1.46	0.72
	Health Post	0.94	0.59–1.49	0.80
Provider sex	Male (Ref.)	—	—	—
	Female	0.92	0.76–1.11	0.40
Previous contact	Yes (Ref.)	—	—	—
	No	2.25	1.86–2.72	<0.001
Marital status	Single (Ref.)	—	—	—
	Married	0.69	0.49–0.98	0.03
	Widowed	1.18	0.34–4.04	0.79
	Divorced	0.97	0.36–2.62	0.95
	Separated	0.34	0.07–1.65	0.19
Region	Afar (Ref.)	—	—	—
	Amhara	0.29	0.17–0.49	<0.001
	Oromia	0.65	0.40–1.03	0.07
	Somali	0.49	0.24–1.02	0.05
	Benishangul-Gumuz	3.77	1.95–7.28	<0.001
	S.N.N. P	0.62	0.37–1.03	0.06
	Gambella	2.96	1.67–5.24	<0.001
	Harari	0.74	0.30–1.80	0.51
	Addis Ababa	0.41	0.23–0.73	<0.001
	Dire Dawa	3.04	1.50–6.15	<0.001
	Sidama	1.04	0.62–1.74	0.89
Residence	Urban (Ref.)	—	—	—
	Rural	0.97	0.79–1.19	0.80

Substantial regional variation persisted after adjustment. Compared with Afar, women attending facilities in Benishangul-Gumuz (AOR = 3.77; 95% CI: 1.95–7.28), Gambella (AOR = 2.96; 95% CI: 1.67–5.24), and Dire Dawa (AOR = 3.04; 95% CI: 1.50–6.15) had higher adjusted odds of receiving FP services during the visit. In contrast, lower odds were observed in Amhara (AOR = 0.29; 95% CI: 0.17–0.49) and Addis Ababa (AOR = 0.41; 95% CI: 0.23–0.73). Confidence intervals for some regions were wider, indicating reduced precision. These adjusted regional estimates reflect conditional associations after accounting for differences in client, provider, and facility characteristics and therefore may differ from the crude descriptive distributions presented in **Table 1**. There were no statistically significant links between educational status, number of pregnancies, type of facility, provider sex, or place of residence.

### Predicted probabilities and marginal effects

**Fig 1:**
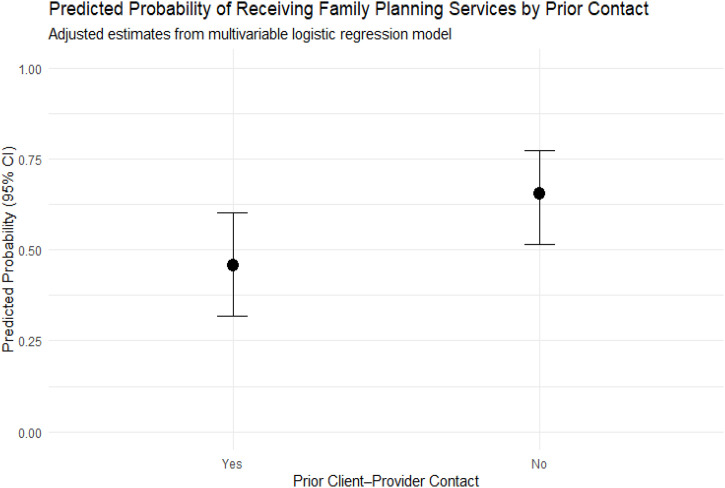
Adjusted predicted probability of family planning service receipt by prior client–provider contact.

**Fig 2:**
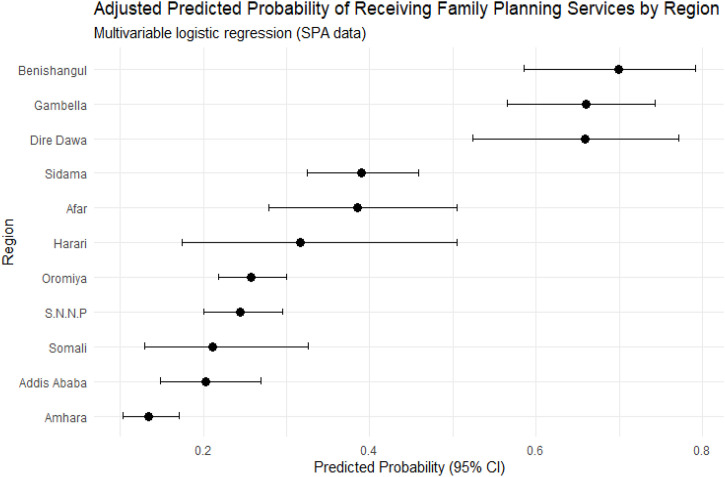
Adjusted predicted probability of family planning service receipt by region.

Predicted probabilities and marginal effects were consistent with regression findings, demonstrating differences in service receipt across prior client–provider contact and region (Fig 1 and Fig 2).

Women without prior contact had a 17.1 percentage point higher probability of receiving FP services compared with those with prior contact (AME = 0.17; 95% CI: 0.132–0.21; p < 0.001). Married women had a 7.8 percentage point lower probability compared with single women (AME = −0.08; 95% CI: −0.15 to −0.00; p = 0.04). Regional marginal effects were directionally consistent with regression estimates (**Table 6**). Average marginal effects were estimated from the fitted multivariable logistic regression model. It was presented to improve interpretability of adjusted probability differences.

**Table 6:** Average marginal effects for predictors of receipt of family planning services during facility visits.

Variable	AME	SE	p-value	95% CI
Previous contact (No vs Yes)	0.17	0.02	<0.001	(0.13, 0.21)
Married vs Single	−0.08	0.04	0.04	(−0.15, −0.00)
Amhara vs Afar	−0.24	0.06	<0.001	(−0.36, −0.13)
Benishangul-Gumuz vs Afar	0.30	0.07	<0.001	(0.16, 0.44)
Dire Dawa vs Afar	0.26	0.08	<0.001	(0.10, 0.41)
Gambella vs Afar	0.25	0.07	<0.001	(0.12, 0.38)

### Interaction effects (Sensitivity analysis)

**Table 7** presents likelihood ratio tests for interaction effects. No evidence of interaction was observed between prior client–provider contact and facility type (χ² = 1.18, df = 3, p = 0.76).

**Table 7:** Likelihood ratio tests for interaction effects on FP service receipt.

Interaction term	χ²	df	p-value
Prior contact × Facility type	1.18	3	0.76
Prior contact × Region	244.93	10	<0.001

Although the likelihood ratio test suggested a statistically significant interaction between prior contact and region (χ² = 244.93, df = 10, p < 0.001), the interaction model produced unstable estimates characterized by inflated standard errors, sparse cell counts, and reduced interpretability. Therefore, interaction terms were not retained, and the main-effects model was selected for final interpretation.

### Model diagnostics and performance

Model diagnostics showed no evidence of problematic multicollinearity or influential observations (S1 Table, S1 Fig, S2 Fig). The model demonstrated acceptable discriminative ability (AUC = 0.72; 95% CI: 0.70–0.74) and satisfactory calibration (Brier score = 0.20) (S3 Table, S3 Fig, S4 Fig). Cross-validation results showed similar raw and adjusted prediction error estimates (S2 Table). Detailed diagnostic results are provided in the Supplementary Material.

## Discussion

This study provides facility-based evidence on family planning (FP) service receipt among women attending health facilities in Ethiopia using nationally implemented Service Provision Assessment (SPA) data. By focusing on FP services delivered during a specific facility visit rather than population-level contraceptive use, the analysis complements household-based evidence and provides insight into service delivery processes occurring during clinical encounters. This distinction is important, as facility-based measures capture whether services are provided during a visit, whereas population-based surveys reflect contraceptive use over time, representing conceptually different outcomes.

A key finding of this study is the inverse association between prior client–provider contact and receipt of FP services during the visit after adjustment for covariates. This contrasts with evidence from population-based studies, where contact with health providers is typically associated with higher contraceptive use [26]. The discrepancy can be explained by differences in outcome definition and timing. In the present study, the outcome reflects service delivery at a single visit rather than cumulative contraceptive behavior.

An important methodological observation in this study was the reversal in direction between the crude and adjusted associations for prior client–provider contact. In unadjusted analysis, women without prior contact had significantly lower odds of receiving FP services during the visit. However, after adjustment for region, facility type, marital status, and other covariates, women without prior contact demonstrated higher adjusted odds of receiving FP services during the visit. The reversal between crude and adjusted estimates suggests that the unadjusted association was strongly confounded by contextual factors, particularly regional and facility-level differences in service delivery patterns. In particular, women with prior contact were disproportionately concentrated within specific regional and facility contexts where the probability of receiving FP services during a given visit differed substantially, thereby altering the conditional association after adjustment.

From a conceptual perspective, prior client–provider contact likely reflects heterogeneous client trajectories rather than a uniform exposure. One possible explanation is that women with prior contact may include returning or continuing users who had previously received counseling or initiated contraception and therefore may have been less likely to require additional FP services during a subsequent visit. In contrast, women with prior contact may represent clients who had previously interacted with FP services and therefore may have been less likely to require additional FP services during the observed visit. This interpretation is consistent with evidence from Ethiopia showing that contraceptive discontinuation and gaps in counseling quality affect how services are used [27–30]. Accordingly, the observed association should not be interpreted as a causal effect, as prior contact may proxy unmeasured characteristics such as prior contraceptive use, service experience, or underlying demand for family planning.

The adjusted association observed in this study also highlights the importance of the content and completeness of services delivered during client–provider interactions. Evidence from Ethiopia indicates that many clients do not receive comprehensive counseling, including information on side effects and alternative methods [31–34]. Service quality improvements have not necessarily accompanied improvements in contraceptive prevalence [32]. These findings suggest that prior contact alone may not be sufficient to ensure FP service delivery during subsequent facility encounters, emphasizing the need to consider both access and quality dimensions of care. Because the SPA client exit interview captures a single encounter rather than longitudinal service trajectories, temporal ordering between prior contact and current service receipt cannot be fully established. Residual confounding by unmeasured factors, including prior contraceptive history, fertility intentions, and reasons for facility attendance, also cannot be excluded.

Substantial regional variation in FP service receipt was observed after adjustment for individual and facility characteristics. Higher adjusted odds of service receipt in Benishangul-Gumuz, Gambella, and Dire Dawa, and lower odds in Amhara and Addis Ababa, indicate persistent contextual disparities. These findings differ from descriptive distributions, where some regions contributed larger proportions of service recipients, highlighting the importance of adjustment for confounding factors when interpreting regional patterns. The persistence of regional differences after adjustment suggests that unmeasured contextual and health-system factors may influence FP service delivery during facility visits [12,35–37]. Although interaction effects were explored to assess potential heterogeneity, unstable estimates due to sparse data limited their interpretability; therefore, regional differences are interpreted at the main-effects level.

Marital status was also associated with service receipt. Married women had lower adjusted odds of receiving FP services during a visit compared with single women, despite representing the majority of clients descriptively. This differs from population-based studies reporting higher contraceptive use among married women [38,39]. This discrepancy is consistent with the distinction between contraceptive use and service receipt at a single visit. One possible explanation is that married women may have differed in prior service experience or reasons for attending the facility, which could influence the likelihood of receiving FP services during a given visit [31].

No statistically significant associations were observed for educational status, number of pregnancies, facility type, provider sex, or place of residence after adjustment. These findings suggest that the study did not identify sufficient evidence of independent associations between these variables and FP service receipt during facility visits within the analyzed sample. However, the absence of statistical significance should be interpreted cautiously because residual confounding, limited variability, or insufficient precision may have influenced these estimates.

Overall, this study contributes to the literature by shifting the focus from population-level contraceptive use to service delivery within health facilities. This perspective reveals how client characteristics, provider interactions, and contextual factors influence whether services are delivered during clinical encounters, offering a more proximal measure of health system performance.

### Limitations

Several limitations should be considered when interpreting the findings. First, the cross-sectional design prevents causal inference, necessitating that all reported associations be regarded as non-causal. Second, the outcome variable receipt of a family planning service during the visit combines multiple service components, including counseling and method provision, which may have distinct determinants. Consequently, the results represent the overall service delivery during a visit rather than particular contraceptive behaviors, including initiation, continuation, or sustained use. Third, the facility-based sample includes only women who attended health facilities offering family planning services, which may introduce selection bias and limits generalizability to the broader population, particularly women who do not access facility-based care. Fourth, the analysis may be affected by endogeneity, particularly for prior client–provider contact, which may proxy unobserved characteristics such as prior contraceptive use, service satisfaction, or underlying demand for family planning. Consequently, the observed association should be interpreted with caution. Fifth, although interaction effects were explored to assess potential effect heterogeneity, they were not retained in the final model due to sparse data across subgroups, inflated standard errors, and instability of parameter estimates. Therefore, the main-effects model may not fully capture contextual variation.

Finally, because analyses were conducted without incorporating SPA sampling weights and complex survey design features, findings should primarily be interpreted as associations within the analyzed SPA facility-visit sample rather than nationally representative population estimates.

## Conclusions and recommendations

Family planning service receipt during facility visits in Ethiopia is associated with prior client–provider contact, marital status, and regional context. The persistence of regional differences after adjustment suggests that contextual differences in service delivery environments may influence FP service receipt during facility visits.

The findings suggest that the presence of prior contact does not necessarily translate into service delivery at subsequent visits, highlighting the importance of the content and timing of client–provider interactions. In addition, substantial regional variation indicates the need to consider context-specific differences in service delivery environments.

**Based on these findings, the following recommendations are proposed:**

- Assess and strengthen the content and completeness of FP-related client–provider interactions, given that prior contact alone was not consistently associated with service receipt during subsequent visits.
- Implement region-specific strategies to address disparities in service delivery, given the substantial variation observed across regions.
- Differentiate client pathways (e.g., first-time versus returning clients) within service delivery and monitoring systems to better align services with client needs.
- Improve measurement of service components in facility-based data systems by distinguishing counseling-only encounters from method provision, given their potentially different determinants observed in this study.

## Supporting information

S1 Table Assessment of multicollinearity among predictors included in the multivariable logistic regression model. (DOCX)

S2 Table Cross-validation estimates of prediction error for the multivariable logistic regression model. (DOCX)

S3 Table Model performance and validation metrics including area under the curve (AUC), 95% confidence interval, Brier score, calibration slope, calibration intercept, and cross-validation error. (DOCX)

S1 Fig Cook’s distance plot for identifying influential observations in the logistic regression model. (DOCX)

S2 Fig Residuals versus leverage plot for the final multivariable logistic regression model. (DOCX)

S3 Fig Receiver operating characteristic (ROC) curve for the fitted logistic regression model. (DOCX)

S4 Fig Calibration plot comparing predicted and observed probabilities of family planning service uptake. (DOCX)

## References

[1] WHO. Family planning a global handbook for providers. 2022.

[2] Kantorová V, Wheldon MC, Ueffing P, Dasgupta ANZ. Estimating progress towards meeting women’s contraceptive needs in 185 countries: a Bayesian hierarchical modelling study. PLoS Med. 2020;17(2):e1003026. doi: 10.1371/journal.pmed.1003026 32069289 PMC7028249

[3] Senderowicz L. Contraceptive autonomy: conceptions and measurement of a novel family planning indicator. Stud Fam Plann. 2020;51(2):161–76. doi: 10.1111/sifp.12114 32358789

[4] Zimmerman LA, Karp C, Kassa M, Lulu B, Yihdego M, Anjur-Dietrich S, et al. What is the relationship between contraceptive services and knowledge of abortion availability and legality? Evidence from a national sample of women and facilities in Ethiopia. Health Policy Plan. 2023;38(3):330–41. doi: 10.1093/heapol/czac103 36440697 PMC10019562

[5] Holt K, Zavala I, Quintero X, Hessler D, Langer A. Development and validation of the client-reported quality of contraceptive counseling scale to measure quality and fulfillment of rights in family planning programs. Stud Fam Plann. 2019;50(2):137–58. doi: 10.1111/sifp.12092 31120147 PMC6618078

[6] Silumbwe A, Nkole T, Munakampe MN, Milford C, Cordero JP, Kriel Y, et al. Community and health systems barriers and enablers to family planning and contraceptive services provision and use in Kabwe District, Zambia. BMC Health Serv Res. 2018;18(1):390. doi: 10.1186/s12913-018-3136-4 29855292 PMC5984360

[7] Kruk ME, Gage AD, Arsenault C, Jordan K, Leslie HH, Roder-DeWan S, et al. High-quality health systems in the Sustainable Development Goals era: time for a revolution. Lancet Glob Health. 2018;6(11):e1196–252. doi: 10.1016/S2214-109X(18)30386-3 30196093 PMC7734391

[8] Merid MW, Kibret AA, Alem AZ, Asratie MH, Aragaw FM, Chilot D, et al. Spatial variations and multi-level determinants of modern contraceptive utilization among young women (15–24 years) in Ethiopia: spatial and multi-level analysis of mini-EDHS 2019. Contracept Reprod Med. 2023;8(1). doi: 10.1186/s40834-023-00224-0 PMC10084619 37038207

[9] Sirage N, Desalegn Z, Wako WG, Yimer A, Bizuneh FK, Feleke SF, et al. Family planning utilization among postpartum women in the Bule Hora District, southern Ethiopia. Front Glob Womens Health. 2024;5:1323024. doi: 10.3389/fgwh.2024.1323024 39717797 PMC11663921

[10] Asmamaw DB, Negash WD. Unmet need for family planning and associated factors among adolescent girls and young women in Ethiopia: a multilevel analysis of Ethiopian Demographic and Health Survey. Contracept Reprod Med. 2023;8(1). doi: 10.1186/s40834-022-00211-x PMC9900907 36740700

[11] Turi E, Mamo Ayana G, Temesgen S, Tafari Shama A, Merga BT, Tolossa T. Determinants of unmet need for contraceptive method among young married women in Ethiopia: Multilevel analysis of Ethiopia Demographic and Health Survey 2016. PLoS One. 2024;19(9):e0306068. doi: 10.1371/journal.pone.0306068 39236003 PMC11376545

[12] Liyeh TM, Dawson A, Mahimbo A, Hayen A. Structural quality of health facilities to provide family planning services in Ethiopia: evidence from the 2021-22 Ethiopia service provision assessment survey. PLOS Glob Public Health. 2025;5(12):e0005377. doi: 10.1371/journal.pgph.0005377 41329758 PMC12671807

[13] Mruts KB, Gebremariam TB, Gebremedhin AT. Predictors of quality of family planning counselling in Ethiopia: multilevel analysis of the SPA survey 2021/22. Front Reprod Health. 2026;7. doi: 10.3389/frph.2025.1743257 PMC12852443 41623699

[14] Tarekegn SM, Tadesse D, Argaw MD, Semahegn A, Taddesse L, Shifarraw S, et al. Capacity and performance of primary health care in Ethiopia: a novel mixed methods measurement in low-income country. BMC Prim Care. 2025;26(1):299. doi: 10.1186/s12875-025-02988-7 41023838 PMC12481810

[15] Solon G, Haider SJ, Wooldridge JM. What are we weighting for?. J Hum Resour. 2015;50(2):301–16. doi: 10.3368/jhr.50.2.301

[16] Beaumont JF. A note on regression estimation in surveys. Biometrika. 2008;95(3):539–53. doi: 10.1093/biomet/asn028

[17] Gelman A. Struggles with survey weighting and regression modeling. Statist Sci. 2007;22(2). doi: 10.1214/088342306000000691

[18] Korn EL, Graubard BI. Analysis of health surveys. New York: Wiley; 1999.

[19] Cohen J. Statistical power analysis for the behavioral sciences. Taylor & Francis Group: Psychology Press. 2009.

[20] Austin PC. Balance diagnostics for comparing the distribution of baseline covariates between treatment groups in propensity-score matched samples. Stat Med. 2009;28(25):3083–107. doi: 10.1002/sim.3697 19757444 PMC3472075

[21] Stuart EA. Matching methods for causal inference: a review and a look forward. Stat Sci. 2010;25(1):1–21. doi: 10.1214/09-STS313 20871802 PMC2943670

[22] Kim JH. Multicollinearity and misleading statistical results. Korean J Anesthesiol. 2019;72(6):558–69. doi: 10.4097/kja.19087 31304696 PMC6900425

[23] Sutherland C, Hare D, Johnson PJ, Linden DW, Montgomery RA, Droge E. Practical advice on variable selection and reporting using Akaike information criterion. Proc Biol Sci. 2023;290(2007):20231261. doi: 10.1098/rspb.2023.1261 37752836 PMC10523071

[24] Onukwugha E, Bergtold J, Jain R. A primer on marginal effects-part II: health services research applications. Pharmacoeconomics. 2015;33(2):97–103. doi: 10.1007/s40273-014-0224-0 25358482

[25] Williams R. Using the margins command to estimate and interpret adjusted predictions and marginal effects. The Stata Journal: Promoting communications on statistics and Stata. 2012;12(2):308–31. doi: 10.1177/1536867x1201200209

[26] Bellow N, Dougherty L, Nai D, Kassegne S, Nagbe RHY, Babogou L, et al. Improving provider and client communication around family planning in Togo: results from a cross-sectional survey. PLOS Glob Public Health. 2023;3(6):e0001923. doi: 10.1371/journal.pgph.0001923 37289680 PMC10249824

[27] Mihretie GS, Abebe SM, Abera M, Assefa DT. An interpretative study of LARCs discontinuation in Ethiopia: the experiences of women accessing contraceptives in selected public health facilities. Open Access J Contracept. 2023;14:41–51. doi: 10.2147/OAJC.S394590 36824684 PMC9942686

[28] Gebeyehu NA, Tegegne KD, Biset G, Sewuyew DA, Alemu BW, Yitayew AM. Discontinuation of long acting reversible contraceptive use and its determinants among women in Ethiopia: Systematic review and meta-analysis. Front Public Health. 2022;10:979231. doi: 10.3389/fpubh.2022.979231 36561863 PMC9763286

[29] Alvergne A, Stevens R, Gurmu E. Side effects and the need for secrecy: characterising discontinuation of modern contraception and its causes in Ethiopia using mixed methods. Contracept Reprod Med. 2017;2:24. doi: 10.1186/s40834-017-0052-7 29201429 PMC5683325

[30] Ayele SG, Mekonnen B, Deribe L, Tsige AW. Prevalence of modern contraceptive discontinuation and associated factors among married reproductive age group women in Debre Berhan town, Ethiopia: a community-based cross-sectional study. BMJ Open. 2024;14(4):e066605. doi: 10.1136/bmjopen-2022-066605 38684273 PMC11086279

[31] Mruts KB, Tessema GA, Dunne J, Gebremedhin AT, Scott J, Pereira GF. Does family planning counselling during health service contact improve postpartum modern contraceptive uptake in Ethiopia? A nationwide cross-sectional study. BMJ Open. 2022;12(5):e060308. doi: 10.1136/bmjopen-2021-060308 35537784 PMC9092163

[32] Hrusa G, Spigt M, Dejene T, Shiferaw S. Quality of family planning counseling in Ethiopia: trends and determinants of information received by female modern contraceptive users, evidence from national survey data, (2014- 2018). PLoS ONE. 2020. doi: 10.1371/journal.pone.0228714 32040485 PMC7010283

[33] Abebaw N, Haile B, Workie A, Mebratu W, Getie M. Quality of family planning counseling and associated factors among reproductive age women who are current contraceptive users at Dessie town health facilities east Amhara. BMC Health Serv Res. 2024;24(1). doi: 10.1186/s12913-024-11833-z 39501390 PMC11536775

[34] Tesfu A, Beyene F, Sendeku F, Wudineh K, Azeze G. Uptake of postpartum modern family planning and its associated factors among postpartum women in Ethiopia: A systematic review and meta-analysis. Heliyon. 2022;8(1):e08712. doi: 10.1016/j.heliyon.2021.e08712 35036604 PMC8753128

[35] Ejigu BA, Seme A, Zimmerman L, Shiferaw S. Trend and determinants of quality of family planning counseling in Ethiopia: evidence from repeated PMA cross-sectional surveys, (2014-2019). PLoS One. 2022;17(5):e0267944. doi: 10.1371/journal.pone.0267944 35622817 PMC9140310

[36] Lee H, Kindane EG, Doh YA, Nam EW. Determinants of modern family planning methods in Ethiopia: a community-based, cross-section mixed methods study. Dialogues Health. 2022;1:100025. doi: 10.1016/j.dialog.2022.100025 38515894 PMC10953940

[37] Gebrekidan H, Alemayehu M, Debelew GT. Individual and community level factors associated with modern contraceptive utilization among women in Ethiopia: multilevel modeling analysis. PLoS One. 2024;19(5):e0303803. doi: 10.1371/journal.pone.0303803 38753736 PMC11098393

[38] Shagaro SS, Gebabo TF, Mulugeta BT. Four out of ten married women utilized modern contraceptive method in Ethiopia: a multilevel analysis of the 2019 Ethiopia mini demographic and health survey. PLoS One. 2022;17(1):e0262431. doi: 10.1371/journal.pone.0262431 35030213 PMC8759669

[39] Hellwig F, Saad GE, Wendt A, Barros AJ. Women’s marital status and use of family planning services across male- and female-headed households in low- and middle-income countries. J Glob Health. 2023;13:04015. doi: 10.7189/jogh.13.04015 36862138 PMC9980282

