# Supplementary material for: Family planning service receipt during facility visits in Ethiopia: Evidence from the 2021–2022 service provision assessment survey
Source: PLoS One. 2026 Jul 9;21(7):e0352145. doi: 10.1371/journal.pone.0352145 (PMC13349127; doi:10.1371/journal.pone.0352145)
Supplement: S1 Table — (DOCX) [file pone.0352145.s001.docx]

**Table S1** summarizes the assessment of multicollinearity among predictors included in the multivariable logistic regression model. No evidence of significant correlation among predictors was observed, with all adjusted generalized variance inflation factor (GVIF) values below commonly accepted thresholds.

**Table S1.** Assessment of multicollinearity among predictors included in the multivariable logistic regression model.

| **Variable** | **GVIF** | **df** | **GVIF^(1/(2×df))** |
| --- | --- | --- | --- |
| Ever attending school | 1.22 | 1 | 1.11 |
| Number of pregnancies | 1.33 | 1 | 1.15 |
| Type of facility | 1.59 | 3 | 1.08 |
| Service provider sex | 1.11 | 1 | 1.06 |
| Previous contact with provider | 1.08 | 1 | 1.04 |
| Marital status | 1.18 | 4 | 1.02 |
| Region | 1.74 | 10 | 1.03 |
| Residence | 1.35 | 1 | 1.16 |
