# Supplementary material for: Family planning service receipt during facility visits in Ethiopia: Evidence from the 2021–2022 service provision assessment survey
Source: PLoS One. 2026 Jul 9;21(7):e0352145. doi: 10.1371/journal.pone.0352145 (PMC13349127; doi:10.1371/journal.pone.0352145)
Supplement: S2 Table — (DOCX) [file pone.0352145.s002.docx]

The logistic regression model demonstrated good discriminative ability, with an area under the receiver operating characteristic (AUC) of 0.724 (Fig S3). Cross-validation results showed similar raw and adjusted prediction error estimates, indicating stable model performance with minimal optimism bias **(Table S2).**

**Table S2.** Cross-validation estimates of prediction error for the multivariable logistic regression model.

| **Estimate** | **Prediction error** |
| --- | --- |
| Raw | 0.2042 |
| Adjusted | 0.2041 |
