# Supplementary material for: Family planning service receipt during facility visits in Ethiopia: Evidence from the 2021–2022 service provision assessment survey
Source: PLoS One. 2026 Jul 9;21(7):e0352145. doi: 10.1371/journal.pone.0352145 (PMC13349127; doi:10.1371/journal.pone.0352145)
Supplement: S3 Table — (DOCX) [file pone.0352145.s003.docx]

Model performance metrics are presented in **Table S3.** The model demonstrated acceptable discriminatory ability, with an area under the receiver operating characteristic curve (AUC = 0.724; 95% CI: 0.704–0.745). The Brier score was 0.201, and the cross-validation error was 0.205, indicating stable predictive performance.

**Table S3.** Model performance and validation metrics

| Metric | Value |
| --- | --- |
| AUC (ROC) | 0.724 |
| 95% CI (AUC) | 0.704 – 0.745 |
| Brier score | 0.201 |
| Calibration slope | 1.00 |
| Calibration intercept | ~0 |
| Cross-validation error | 0.205 |
