# Supplementary material for: Family planning service receipt during facility visits in Ethiopia: Evidence from the 2021–2022 service provision assessment survey
Source: PLoS One. 2026 Jul 9;21(7):e0352145. doi: 10.1371/journal.pone.0352145 (PMC13349127; doi:10.1371/journal.pone.0352145)

Diagnostic plots based on residuals versus leverage indicated no influential observations affecting model estimates **(Fig S2).**

**Fig S2**. Residuals versus leverage plot for the final multivariable logistic regression model**.**
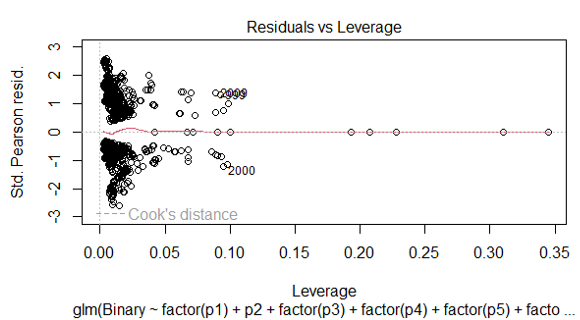

Supplement: S2 Fig — (DOCX) [file pone.0352145.s005.docx]
