# Supplementary material for: Family planning service receipt during facility visits in Ethiopia: Evidence from the 2021–2022 service provision assessment survey
Source: PLoS One. 2026 Jul 9;21(7):e0352145. doi: 10.1371/journal.pone.0352145 (PMC13349127; doi:10.1371/journal.pone.0352145)

**Fig S3** presents the receiver operating characteristic (ROC) curve assessing the discriminatory ability of the fitted logistic regression model. The area under the curve (AUC = 0.724) indicates acceptable discrimination, suggesting that the model reasonably distinguishes between clients who did and did not receive family planning services during the visit.

**Fig S3.** Receiver operating characteristic (ROC) curve for the fitted logistic regression model.


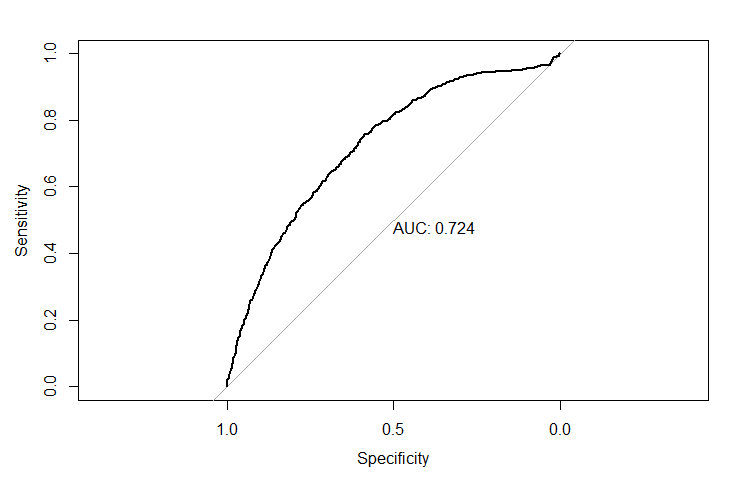

Supplement: S3 Fig — (DOCX) [file pone.0352145.s006.docx]
