# Supplementary material for: Family planning service receipt during facility visits in Ethiopia: Evidence from the 2021–2022 service provision assessment survey
Source: PLoS One. 2026 Jul 9;21(7):e0352145. doi: 10.1371/journal.pone.0352145 (PMC13349127; doi:10.1371/journal.pone.0352145)

**Fig S4** presents the calibration of the logistic regression model, comparing predicted probabilities with observed outcomes. The calibration curve demonstrates good agreement overall, with minor deviations at higher predicted probabilities, indicating that the model predictions are reasonably well calibrated**.**

**Fig S4.** Calibration plot comparing predicted and observed probabilities of family planning service uptake.
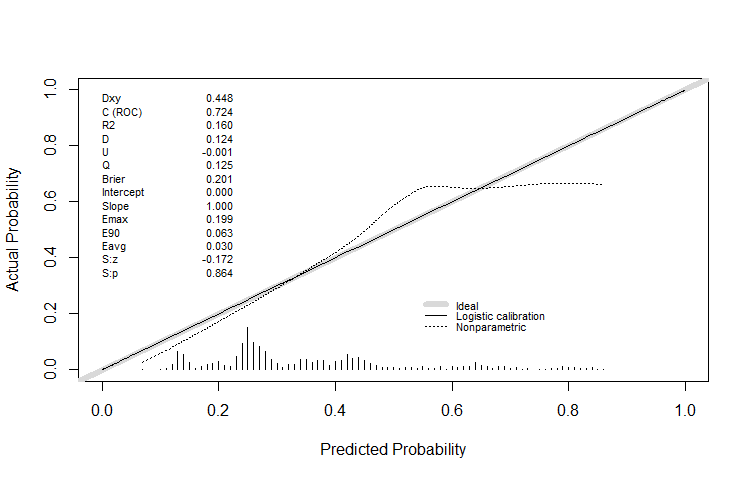

Supplement: S4 Fig — (DOCX) [file pone.0352145.s007.docx]
